# Acute effects of plyometric conditioning activities on sprint performance in sports individuals: a systematic review and meta-analysis

**DOI:** 10.3389/fphys.2026.1793051

**Published:** 2026-03-23

**Authors:** Hangxin Chen, Dongxu Gao

**Affiliations:** 1 School of Physical Education, Shanghai University of Sport, Shanghai, China; 2 Division of Sports Science and Physical Education, Tsinghua University, Beijing, China

**Keywords:** acute effect, conditioning activity, PAPE, plyometric, sprint performance

## Abstract

**Objective:**

Plyometric conditioning activities are widely used in warm-ups to elicit post-activation performance enhancement (PAPE), yet their acute effects on sprint performance and the factors shaping these responses remain unclear. This systematic review and meta-analysis aimed to quantify the acute effects of plyometric conditioning activities on sprint performance in sports individuals, and to identify key moderators of these effects, including conditioning type (single *vs*. complex protocols), rest-interval duration, and sprint test distance (≤30 m *vs*. >30 m).

**Methods:**

A systematic search was conducted across four electronic databases. The methodological quality of the included studies was assessed in accordance with the Cochrane guidelines. Outcome measures were analyzed using R software (version 4.3.0, R studio). A total of fourteen studies involving 198 participants(78.8% male) were included, comprising randomized controlled trials (RCTs), parallel-group trials, crossover trials, and pre–post-test experimental designs. All effect sizes were derived exclusively from within-condition pre–post comparisons.

**Results:**

Plyometric conditioning activities elicited a moderate acute improvement in sprint performance [SMD = 0.51, 95% CI (0.24, 0.79), P < 0.001], with moderate heterogeneity (I²= 51%). Subgroup analyses showed that complex protocols tended to produce larger effects than single protocols, although heterogeneity was substantial for complex training (I²= 80%). Longer rest intervals were associated with significant performance gains [SMD = 0.42, 95% CI (0.16, 0.68), P = 0.002]. Sprint distance moderated the effect: benefits were significant for ≤30 m tests [SMD = 0.73, 95% CI (0.34, 1.13), P = 0.0003], but not for >30 m tests [SMD = 0.17, 95% CI (−0.15, 0.49), P = 0.31], with a significant subgroup difference (P = 0.03).

**Conclusion:**

Plyometric conditioning activities were associated with acute changes in sprint performance, with statistically significant pooled effects observed in shorter sprint distances (≤30 m). Larger pooled effect sizes appeared in complex protocols and longer rest intervals, although responses varied across studies. These findings suggest that protocol characteristics and recovery duration may influence acute sprint performance outcomes.

## 1 Introduction

In high-performance sport, competitive outcomes are often determined by peak displacement velocity achieved at critical moments. In real match play, many decisive actions occur after short bouts of high-speed locomotion and rapid acceleration. For example, analyses of goal-scoring situations in professional soccer have identified linear sprinting as the most frequent antecedent action (Faude et al., 2012). Similarly, in National Rugby League matches, 67.5% of sprints are shorter than 20 m, with 6–10 m sprints being the most common (Gabbett, 2012). Beyond these examples, short accelerations and brief sprint efforts are also central to decisive plays in a range of sports (e.g., basketball fast breaks, field hockey transitions, and racquet-sport court coverage), highlighting the broad relevance of short-distance speed. In parallel, the quantification of acceleration events has been regarded as a key indicator for evaluating external load and performance in team sports, and its reliability and practical utility directly influence training monitoring and pre-competition decision-making (Delaney et al., 2018). Collectively, these findings highlight a highly applied and performance-relevant question: in many sports, winning is less dependent on whether an athlete can reach maximal speed, and more dependent on whether they can enter high speed more rapidly.

Pre-competition warm-up is essential for stable, near-optimal performance early in competition, acting through integrated physiological and psychological pathways in which increased muscle temperature is a central driver of readiness (Bishop, 2003a, Bishop, 2003; McGowan et al., 2015; Saltin et al., 1968). However, real-world constraints often force a trade-off between “warm-up decay” and “warm-up overload” (Bishop, 2003), making 5–20 m acceleration optimization a competition-day readiness problem under limited time and resources.

In response to these constraints, “conditioning activities” (CAs) have gained substantial attention as a refinement of traditional warm-up (Karabel and Makaracı, 2025). Sale defined post-activation potentiation (PAP) as a transient enhancement of contractile performance following prior activity (Sale, 2002), with proposed contributors including myosin regulatory light chain phosphorylation and enhanced neural drive (Hodgson et al., 2005; Tillin and Bishop, 2009). Because potentiation and fatigue coexist, the net outcome is context-dependent and determined by their interaction (Rassier and Macintosh, 2000), which underpins applied recommendations on feasible CA selection, dosing, and recovery windows (Docherty and Hodgson, 2007; Robbins, 2005). To improve conceptual precision and comparability, researchers distinguish muscle-level “PAP” from performance-level “post-activation performance enhancement” (PAPE) (Blazevich and Babault, 2019; Prieske et al., 2020; Zimmermann et al., 2020). Evidence further suggests PAPE in trained athletes may not be strictly localized to a single muscle group (Cuenca-Fernandez et al., 2017), reinforcing the need to quantify CA-specific net sprint effects and identify key moderators.

Among the wide range of CAs, plyometric conditioning activities (e.g., drop/depth jumps, repeated jumps, hurdle jumps, and bounding) are often viewed as particularly promising “field-friendly” priming strategies because they are performed at high movement velocities, are dominated by the stretch–shortening cycle (SSC), closely resemble sprint kinetic demands, and require minimal equipment (Markovic and Mikulic, 2010). This practical appeal has also contributed to their growing attention as warm-up extensions in applied settings (Karabel and Makaracı, 2025). From a long-term training perspective, plyometric training can meaningfully improve lower-limb explosive performance and vertical jump outcomes (Markovic, 2007; Markovic and Mikulic, 2010), and has been associated with systematic improvements in sprint performance (Saez de Villarreal et al., 2012), thereby supporting its rationale as a short-duration pre-competition stimulus. Nevertheless, acute findings remain inconsistent. Some studies report that incorporating plyometric CAs during warm-up improves 20–40 m sprint performance (Creekmur et al., 2017), or that adding loaded jump stimuli to standard sprint warm-ups enhances 10–30 m split times (Tomlinson et al., 2020). Other work suggests that individualized depth-jump protocols can improve 5–20 m acceleration performance across recovery windows ranging from 15 s to 12 min (Byrne et al., 2020), yet marked inter-individual and sport-specific variability persists, and different depth-jump protocols can elicit divergent responses across athletic populations (Dello Iacono et al., 2016). In applied settings, such uncertainty often forces coaching staff to rely on experience and trial-and-error, and when recovery is insufficient, “priming” may shift toward an additional fatigue burden rather than a net benefit (Rassier and Macintosh, 2000). For high-level teams that must make rapid and reproducible competition-day decisions, fragmented and heterogeneous evidence is inadequate.

Despite rapid growth in this research area, important synthesis-level gaps remain and can be framed as a clear unresolved problem. First, many existing systematic reviews and meta-analyses pool heterogeneous CA modalities (e.g., resistance, isometric, plyometric, and resisted sprinting) or combine multiple task domains (e.g., jumps, throws, and other power tasks), which may obscure the true magnitude and potential dose–response characteristics of plyometric CAs on sprint performance—arguably the most frequently used priming approach in field practice (Dobbs et al., 2019; Wilson et al., 2013). Second, meta-analyses focusing on plyometric training effects on jumping and sprinting have primarily examined training interventions or multi-outcome performance changes, and therefore do not directly address the key competition-day decision problem in acute priming: what is the net acute effect on sprint performance across phases and distances, and which protocol characteristics are most likely to yield facilitation rather than fatigue? Moreover, which athletic populations are most likely to benefit remains insufficiently resolved (Xie et al., 2024). Given the performance value of short-distance sprint advantages in modern sport and the real-world constraints on warm-up resources, a targeted quantitative synthesis of the acute effects of plyometric conditioning on sprint performance has clear scientific novelty and direct translational relevance.

Accordingly, we conducted a systematic review and meta-analysis to quantify the acute effects of plyometric conditioning activities on sprint performance in sports individuals and to examine key moderators, including sprint distance (≤30 m *vs*. >30 m), CA format (single *vs*. complex), plyometric vector orientation (vertical *vs*. horizontal), and recovery window characteristics. We hypothesized greater and more consistent benefits in shorter sprint tests (acceleration phase), with protocol format and recovery substantially moderating the net effect. This work aims to provide more actionable evidence to guide competition-day warm-up priming and to inform future PAPE study design and reporting.

## 2 Methods

### 2.1 Experimental approach to the problem

The study protocol was registered in PROSPERO (CRD420251272192).The literature search was carried out using the following online databases: PubMed, Scopus, Embase, and Web of Science. It included studies published until December 27, 2025. Keywords were defined based on previous studies and aligned with the study objectives. The following keywords were used in combination with the Boolean operators “AND” and “OR,” using the PICOs method (ie, participants, intervention, comparator, and outcomes) as part of the search strategy (plyometric* OR “drop jump*” OR “depth jump*” OR “reactive jump*” OR bounding OR “stretch-shortening cycle” OR SSC) AND (“post-activation potentiation” OR “post activation potentiation” OR PAP OR “post-activation performance enhancement” OR “post activation performance enhancement” OR PAPE ORpriming OR “conditioning activity”) AND (sprint* OR accelerat* OR “sprint performance” OR “sprint time” OR “running speed” OR “maximal sprint*”).Lastly, the reference lists from relevant articles were examined to identify other potentially eligible studies.

### 2.2 Inclusion and exclusion criteria

This review considered peer-reviewed English-language studies and dissertations. Inclusion criteria were developed according to the PICOS principle: P (Population) regularly trained healthy individuals, including physically active, amateur athletes, and professional athletes; I (Intervention) Different forms of plyometric conditioning activities; C (Comparison) the within-condition pretest in the plyometric CA; O (Outcome) sprint time and velocity over various distances (e.g., 0–5 m, 0–10 m); S (Study design) randomized controlled, parallel, crossover, and pre–post-test trials.

**Figure 1:**
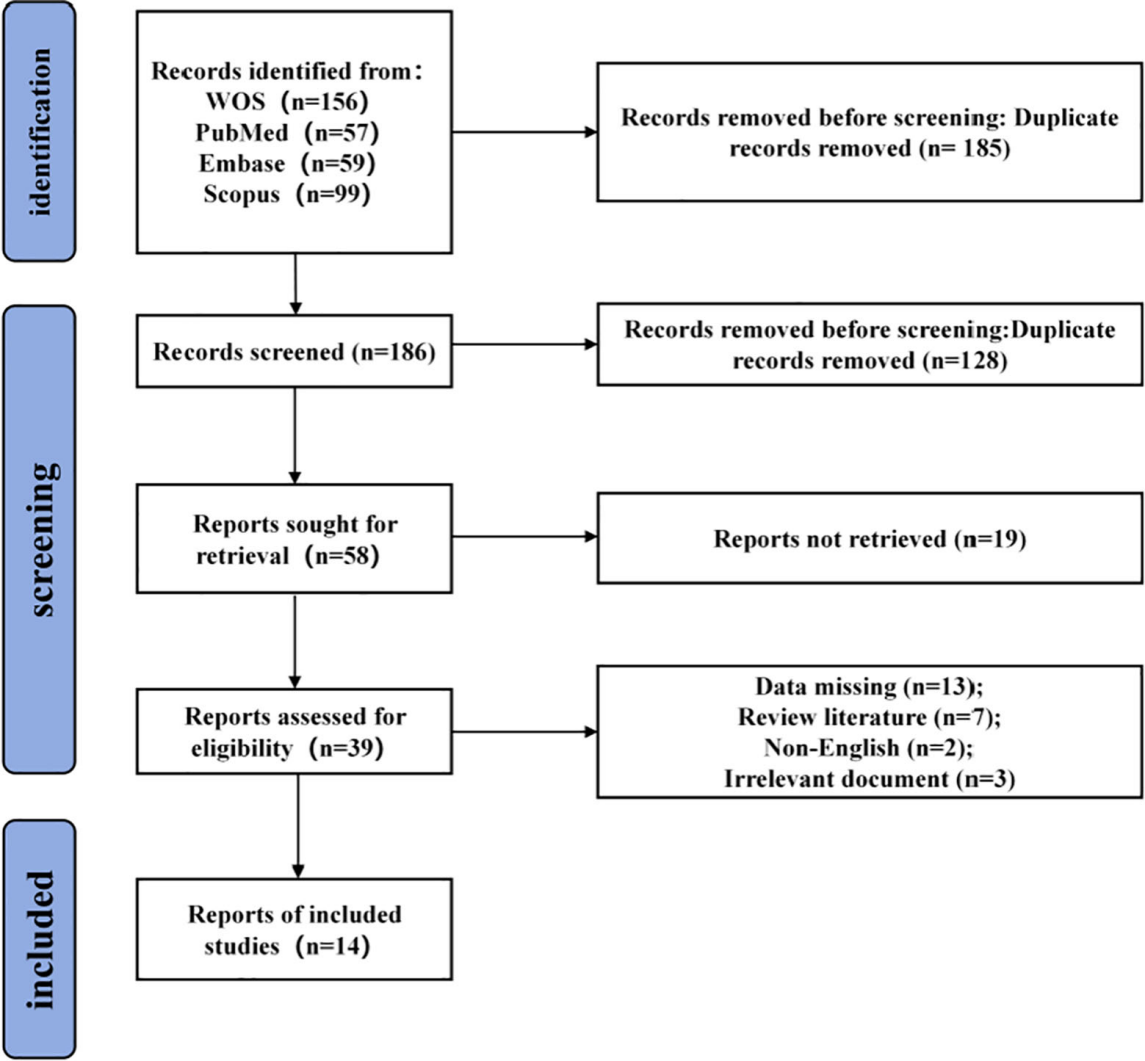
Literature screening flow chart.

Excluded literature consisted of the following: duplicate articles, review articles, interventions other than PAP/PAPE training, studies with missing data, participants with less than 1 year of sports experience, and non-English literature. Please refer to the **Figure 1** for the specific process.

### 2.3 Assessment of methodological quality and risk of bias

Two reviewers (G-DX and C-HX) independently assessed randomized studies using the PEDro scale, evaluating criteria such as random allocation, concealed allocation, baseline between-groups similarity, subjects blinding, therapists blinding, assessors blinding, dropouts, intention-to-treat analysis, between-groups statistical comparison, point measures, and variability data. Eleven criteria were scored 0-1 (0 = no, 1 = yes), excluding criteria 1 and 6 from the review, resulting in a score range of 0-9. The risk of bias for the pre-post-tests was evaluated using the ROBINS-I tool, categorizing bias as “low risk”, “moderate risk,” “serious risk,” “critical risk,” or “no information.”

### 2.4 Data extraction and analysis

Two independent reviewers extracted data from the included studies using a standardized data extraction form. Data were extracted after fulltext review. For data presented in graphs or figures that could not be directly extracted, the GetData Graph Digitizer software was used. Extracted data included: first author, year of publication, sample size, age, potentiation intervention, potentiation load, rest interval, sprint time. All effect sizes were derived exclusively from within-condition pre–post comparisons, and control-group data were not incorporated into the effect size computation.

The differences between premeasurements and post measurements are expressed as standardized mean differences (SMD) with their respective 95% confidence interval (CI). SMDs were used because sprint performance was assessed across a range of distances. The thresholds used to qualitatively interpret SMD were <0.2 (trivial), ≥0.2 (small), ≥0.5 (moderate), and ≥0.8 (large). Heterogeneity among studies was assessed using I2 statistics. I values range between 0% and 100% and are considered low, modest, or high for <25%, 25% (50%), and >50%, respectively. High heterogeneity indicates substantial variability among studies in terms of outcomes and methodological aspects, resulting in varying weights of evidence. While it is not a requirement for conducting a meta-analysis, it is always preferable to have lower levels of heterogeneity among the included studies. Heterogeneity was assumed when the P value of the I² test was < 0.05; statistical significance was set at P < 0.05.

### 2.5 Data synthesis

Analyses were performed using R software (version 4.3.0, R studio). Effect sizes (ES), corrected for small sample sizes using Hedges’ and Olkin’s g, were calculated using the following formula (Equation 1):

(1) $$\text{ES}=\frac{\left(M_{\text{post}}-M_{\text{pre}}\right)}{{\text{SD}}_{\text{pooled}}}\times (1-\frac{3}{4(n_{1}+n_{2}-2)-1})$$

where M_pre_ is the mean of the pretest sprint performance, M_post_ is the mean of the post-test sprint performance, n_1_ and n_2_ are the sample sizes, and SD_pooled_ is the pooled SD (Equation 2):

(2) $${\text{SD}}_{pooled}=\frac{({\text{n}}_{1}-1)\times {\text{SD}}_{1}^{2}+({\text{n}}_{2}-1)\times {\text{SD}}_{2}^{2}}{\left({\text{n}}_{1}+{\text{n}}_{2}-2\right)}$$

where $SD_{1}^{2}$ and $SD_{2}^{2}$ are the SD of the performance test completed before and after the conditioning activity, respectively.

For sprint results reported at the average velocity, the conversion was performed by distance/time. Visualizations were performed using the ggplot2 package and the metafor package. A random-effects model was used for pooling the main results. Publication bias was assessed by visual inspection of funnel plots and the Egger regression test. The Grading of Recommendations Assessment, Development, and Evaluation (GRADE) methodology was used to assess the level of evidence, initially rated as high and downgraded based on sample size, I^2^ > 50%, lack of clear merger direction, and risk of publication bias (**Table 1**).

**Table 1:** GRADE assessment for the certainty of evidence*.

Certainty assessment						No. of participants			Certainty
No. of studies	Risk of bias	Inconsistency	Indirectness	Imprecision	Publication bias	Pre	Post	Absolute (95% CI)	
SPT									
13	not serious	not serious	not serious	not serious	not serious	161	161	ES 0.34 (0.11 to 0.56)	⨁⨁⨁⨁ High
CPT									
4	not serious	not serious	not serious	not serious	not serious	69	69	ES 1.07 (0.22 to 1.92)	⨁⨁⨁⨁ High

*SPT, single plyometric training; CPT, complex plyometric training.

## 3 Results

### 3.1 Study characteristics

According to the PRISMA reporting guidelines, 14 studies were included in the analysis. A total of 198 participants were included, including 156 males (78.8%), 17 females (8.6%), and 25 participants with unreported sex (12.6%). All participants had at least one year of training experience and were part of an athletic population (**Table 2**).

**Table 2:** Included literature information table.

Study	Study designs	Sample size	Conditioning activity (CA)	Forms of intervention		Interval time (min)			Volume (set)		Test distance
				Single	Complex	0.3-4	0.3-5	≥8	1	≥2	
Zimmermann et al.(2021)	RCT crossover	12	3*5(CMJ)	✓		2, 4	2, 5	8, 10		✓	30m
Tomlinson et al. (2020)	Pre-Posttest	22	2*8 (loaded squat jumps) 13% BW	✓			5			✓	30m
Yoshimoto et al.(2016)	RCT crossover	10	3*10 hurdles (height 22 cm , spaced 90 cm apart)	✓				10		✓	60 m
Yoshimoto et al.(2016)	RCT crossover	10	3*60 m(bounding jump)	✓				10		✓	60 m
Pereira et al.(2022)	RCT crossover	12	2*5(Drop-jumps 60 cm)	✓				15		✓	60m
Bomfim Lima et al.(2011)	RCT crossover	10	2*5(Drop-jumps 75 cm)	✓				10, 15		✓	50m
Brink et al.(2022)	RCT parallel	23	3*10(alternate leg weighted bounding)10% BW	✓		2	3			✓	20m
Gil et al.(2019)	Pre-Posttest	11	8*20m(special drills)+ 2*40 m sprints + 2*5(Drop-jumps 70 cm)		✓			15		✓	100m
Creekmur et al. (2017)	RCT crossover	10	2*8(weighted squat jumps)11.3Kg	✓				8		✓	40m(0-20m)
Vanderka et al.(2016)	RCT crossover	12	2*6(half-squat jumps)70.8 ± 19.3 kg	✓		4	5			✓	40m(0-20, 20-40)
Sharma et al.(2018)	RCT crossover	14	2*10(ankle hops)+ 3*5(hurdle hops 70 cm)+ 1*5(drop jumps 50 cm)		✓	1	2	10		✓	20m
Munshi et al.(2022)	RCT crossover	24	5*10 (double-legged vertical)+2*15 m (broad jumps)+1*30 m (single and double legged bounding) +1*5 (depth jumps)		✓	4	5	12		✓	20m
Piper et al.(2020)	RCT crossover	13	3*5(Weighted jump)10% BW	✓		20s, 4	5	8, 12, 16, 20		✓	20m(0–10, 10–20, 0–20)
Haris et al.(2021)	Pre-Posttest	20	2*10(Ankle hops)+ 3*5(Hurdle hops 70 cm)+1*5(Drop jumps)		✓	1	2			✓	20m
Kümmel et al.(2016)	RCT crossover	5	10 reactive hops	✓				10	✓		30m(0-10, 0-20, 0-30)

### 3.2 Methodological quality and risk of bias

**Figure 2:**
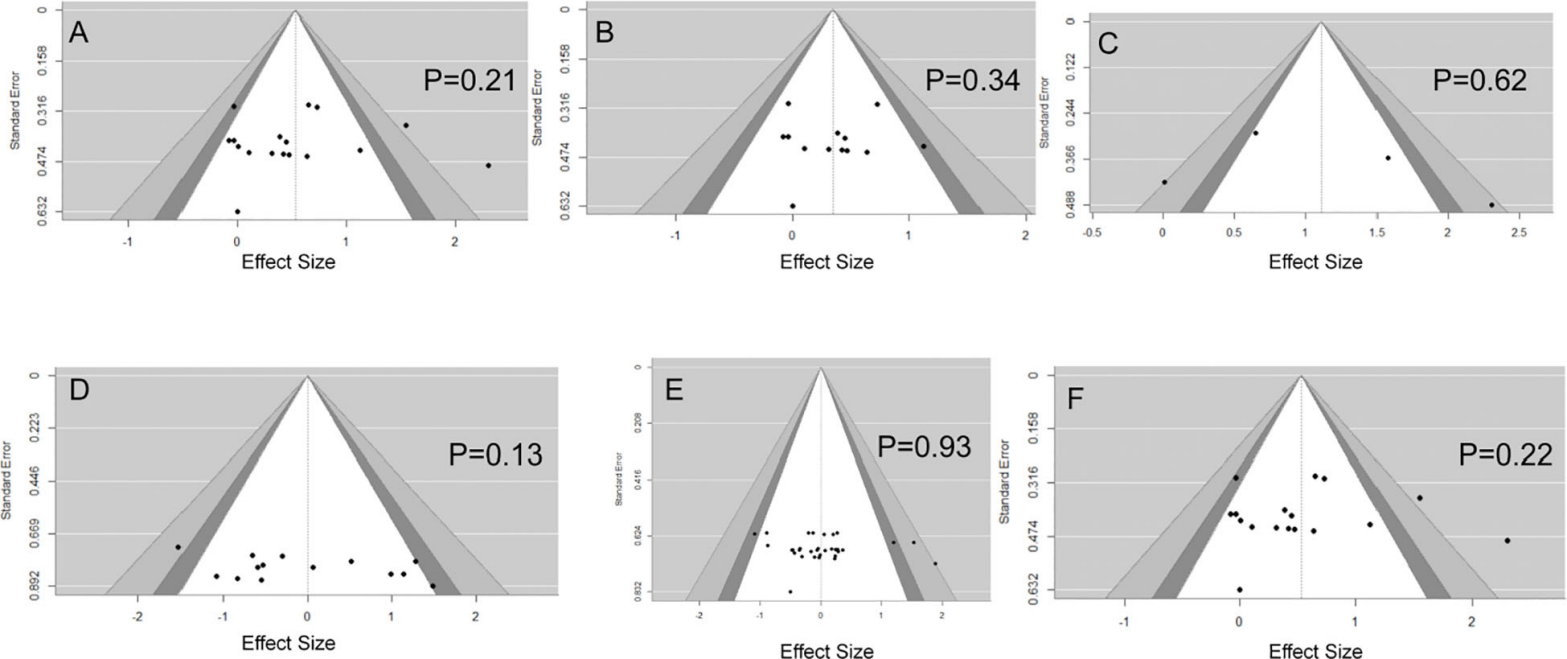
Funnel diagram of publication bias in the included literature. Six funnel plots labeled A to F display effect size on the x-axis and standard error on the y-axis, each showing black data points, shaded significance regions, and corresponding p-values: **(A)** (**Figure 3**): P = 0.21, **(B)** (**Figure 4**): P = 0.34, **(C)** (**Figure 5**): P = 0.62, **(D)** (**Figure 6**): P = 0.13, **(E)** (**Figure 7**): P = 0.93, **(F)** (**Figure 8**): P = 0.22.

The PEDro scores (**Table 3**) for randomized trials ranged from 4 to 6, indicating good methodological quality. Three pre–post-test studies had a low risk of bias. Detailed results of the PEDro and ROBINS-I evaluations (**Table 4**) are provided in the Supplemental Digital Content. The funnel plot exhibited symmetry, and the Egger regression test (p>0.05) indicated no significant publication bias (**Figure 2**).

**Table 3:** Physiotherapy Evidence Database (PEDro) scale.

Study	PEDro item number											Score
	1*	2	3	4	5	6*	7	8	9	10	11	
Zimmermann et al.(2021)	–	0	0	1	0	–	0	1	1	1	1	5
Tomlinson et al. (2020)	–	0	1	0	0	–	0	1	1	1	1	5
Yoshimoto et al.(2016)	–	0	0	1	0	–	0	1	1	1	1	5
Yoshimoto et al.(2016)	–	0	0	0	0	–	0	1	1	1	1	4
Pereira et al.(2022)	–	0	0	1	0	–	0	1	1	1	1	5
Bomfim Lima et al.(2011)	–	0	0	1	0	–	0	1	1	1	1	5
Brink et al.(2022)	–	0	0	1	0	–	0	1	1	1	1	5
Gil et al.(2019)	–	0	0	1	0	–	0	1	1	1	1	5
Creekmur et al. (2017)	–	0	0	1	0	–	0	1	1	1	1	5
Vanderka et al.(2016)	–	0	0	1	0	–	0	1	1	1	1	5
Sharma et al.(2018)	–	0	0	1	0	–	0	1	1	1	1	5
Munshi et al.(2022)	–	0	0	1	0	–	0	1	1	1	1	5
Piper et al.(2020)	–	1	0	1	0	–	0	1	1	1	1	6
Haris et al.(2021)	–	0	0	1	0	–	0	1	1	1	1	5
Kümmel et al.(2016)	–	0	0	1	0	–	0	1	1	1	1	5

*Not included in methodological quality scoring; 1, criterion was satisfied;”-”, criterion was not satisfied. Each satisfied criterion measure, excluding item 1 and 6, contributes 1 point to the total PEDro score (1–9). Criteria, (1) eligibility criteria were specified (*not applicable); (2) random allocation; (3) concealed allocation; (4) groups similar at baseline;(5) blinding of participants; (6) blinding of therapists who administered the therapy (*not applicable); (7) blinding of assessors;(8) less than 15% drop-outs; (9) intention to treat;(10) between-group statistical analysis; (11) point measures and variability data.

**Table 4:** Assessment of the risk of bias in non-randomized studies of interventions*.

Study	Bias due to confounding	Bias in selection of study participants	Bias in measurement classification of intervention	Bias due to deviations from intended interventions	Bias due to missing data	Bias in measurement of outcomes	Bias in selection of reported results
Haris	Low	Low	Moderate	Moderate	Moderate	Low	Low
Mh	Low	Low	Moderate	Moderate	Moderate	Low	Low
Tomlinson	Low	Low	Moderate	Moderate	Moderate	Low	Low

*ROBINS-I, Risk of Bias in Nonrandomized Studies of Interventions. The categories for risk of bias for each domain are “low risk,” “moderate risk,” “serious risk,” and “critical risk” of bias and “no information”.

### 3.3 Acute influence of plyometric training on sprint performance

**Figure 3:**
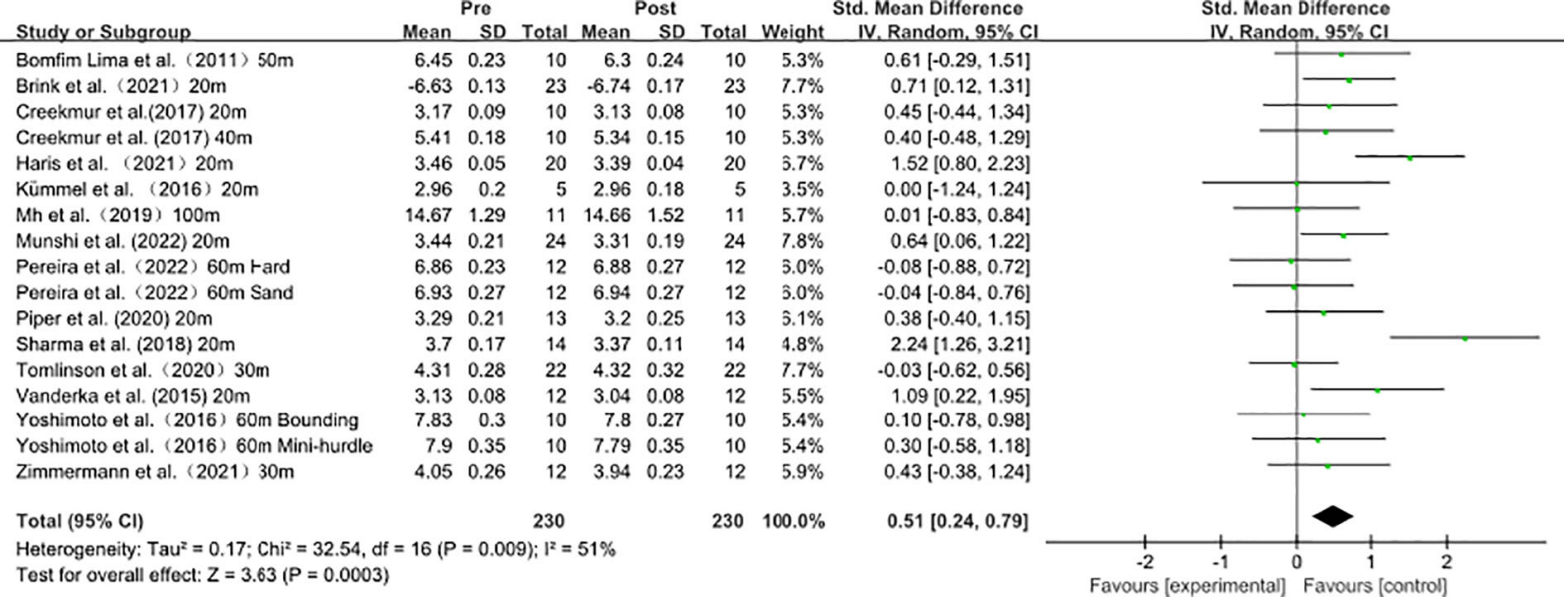
Acute influence of plyometric training on sprint performance.

As shown in **Figure 3**, data from 14 studies (17 effect sizes) demonstrated the acute effects of plyometric conditioning activities on sprint performance. Heterogeneity analysis revealed moderate statistical heterogeneity (I² = 51% > 40%, P = 0.007), indicating significant variability among the included effect sizes. Therefore, a random-effects model was used for the meta-analysis of effect sizes, as shown in the figure. The pooled effect size |SMD| = 0.51 ≥ 0.5 indicates a moderate effect, suggesting that plyometric conditioning activities induce an acute improvement in sprint performance [SMD = 0.51, 95% CI (0.24, 0.79), P < 0.001]. However, given the observed heterogeneity, further subgroup analyses (e.g., sprint distance ≤30 m *vs*. >30 m) are warranted to explore potential moderators.

### 3.4 Acute influence of single plyometric training on sprint performance

**Figure 4:**
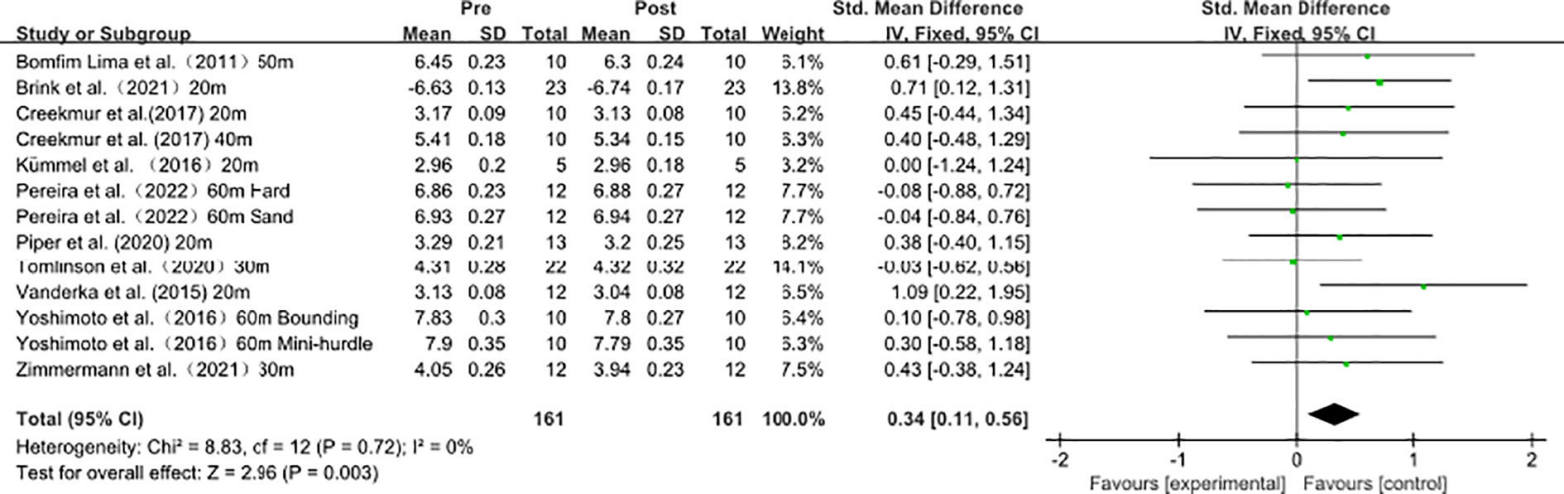
Acute influence of single plyometric training on sprint performance.

As shown in **Figure 4**, the data from 10 studies (13 effect sizes) demonstrated the effects of single plyometric training on sprint performance. Heterogeneity analysis revealed no statistical heterogeneity (I² = 0 ≤ 40%, P = 0.672), indicating the absence of significant heterogeneity among the studies. Therefore, a fixed-effects model was used for the meta-analysis of effect sizes, as shown in the figure. The combined effect size |SMD| = 0.34 > 0 indicates a small effect size, suggesting that single plyometric training induces a slight improvement in sprint performance [SMD = 0.34, 95% CI (0.11, 0.56), P = 0.003].

### 3.5 Acute influence of complex plyometric training on sprint performance

**Figure 5:**
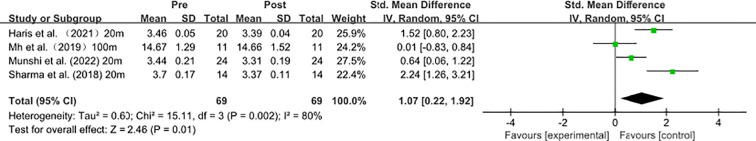
Acute influence of complex plyometric training on sprint performance.

As shown in **Figure 5**, the data from 4 studies (4 effect sizes) demonstrated the effects of complex plyometric training on sprint performance. Heterogeneity analysis revealed high statistical heterogeneity (I² = 80% > 40%, P = 0.002), indicating significant variability among the included effect sizes. Therefore, a random-effects model was used for the meta-analysis of effect sizes, as shown in the figure. The combined effect size |SMD| = 1.07 ≥ 0.8 indicates a large effect size, suggesting that complex plyometric training induces an acute improvement in sprint performance [SMD = 1.07, 95% CI (0.22, 1.93), P = 0.014].

### 3.6 Acute influence of complex plyometric training on sprint performance under different rest intervals

**Figure 6:**
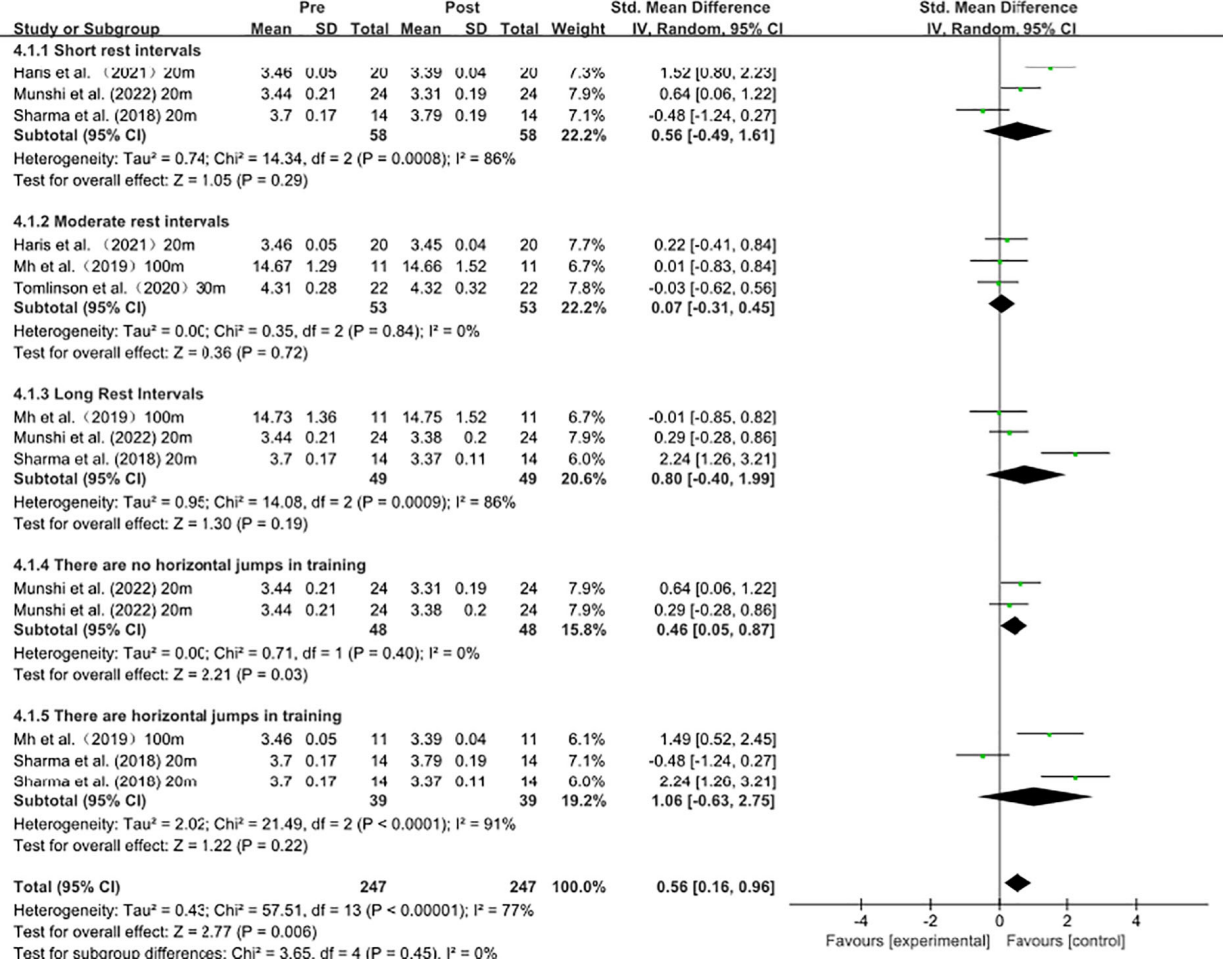
Acute influence of complex plyometric training on sprint performance under different rest intervals.

As shown in **Figure 6**, subgroup analyses suggest that the inclusion of horizontal jump exercises may contribute to between-study heterogeneity. Studies without horizontal jump exercises demonstrated high homogeneity (I² = 0%) and a moderate, statistically significant effect on sprint performance (SMD = 0.46, 95% CI 0.05–0.87). In contrast, studies including horizontal jump exercises exhibited substantial heterogeneity (I² = 91%) and a larger but non-significant pooled effect (SMD = 1.06, 95% CI −0.63–2.75).

Regarding rest intervals, short and long rest subgroups both showed considerable heterogeneity (I² = 86%), with non-significant pooled effects (short: SMD = 0.56; long: SMD = 0.80). In contrast, the moderate-rest subgroup showed no heterogeneity (I² = 0%) but also no significant effect (SMD = 0.07). These findings indicate that rest-interval stratification alone does not adequately explain the observed heterogeneity.

### 3.7 Acute influence of plyometric training on sprint performance under different rest intervals

**Figure 7:**
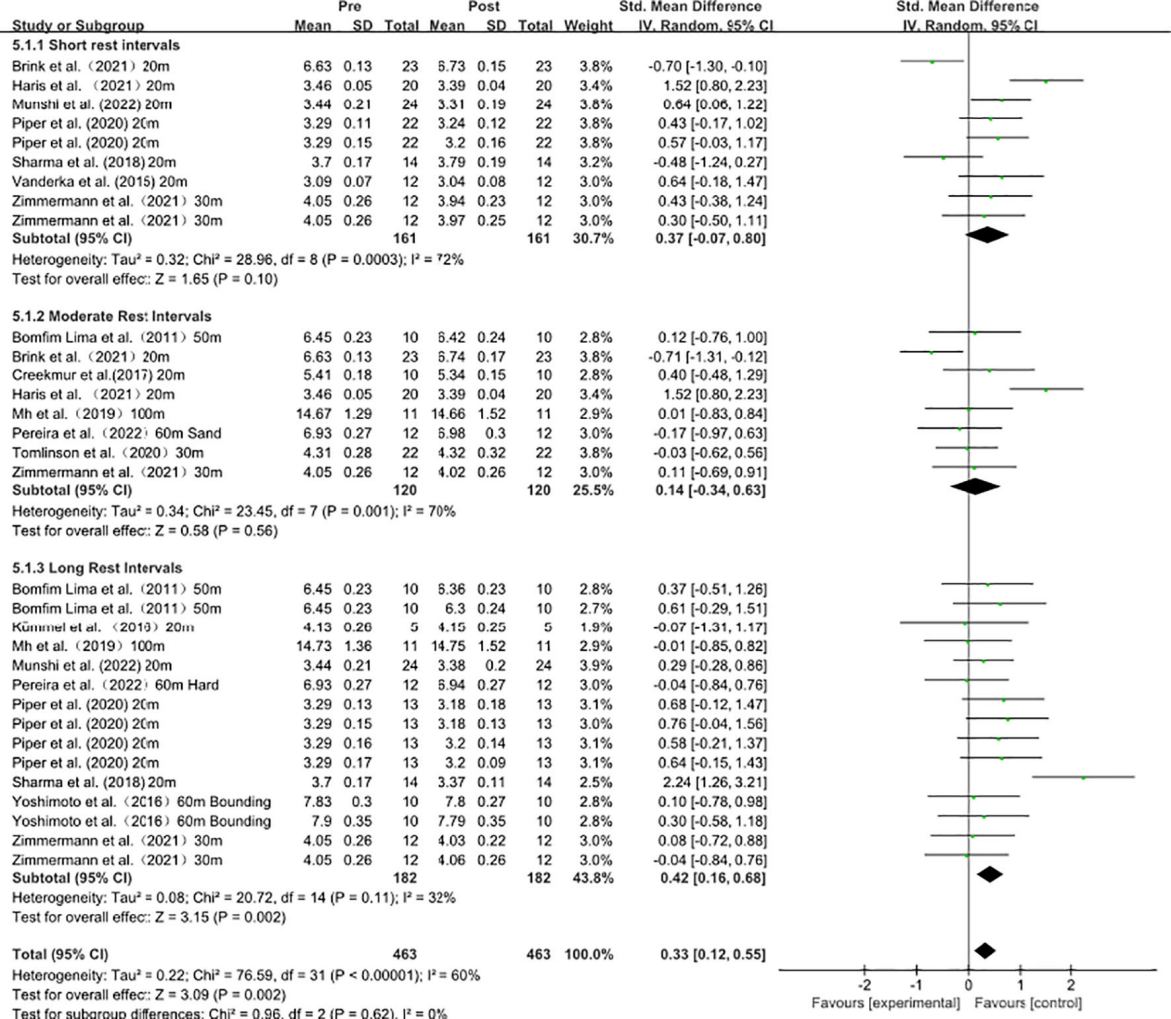
Acute influence of plyometric training on sprint performance under different rest intervals.

As shown in **Figure 7**, subgroup analyses were performed to examine whether rest interval duration moderates the effects of plyometric training on sprint ability. In the short-rest subgroup (9 effect sizes), heterogeneity was high (I² = 72%, P = 0.0003); therefore, a random-effects model was applied. The pooled effect showed a non-significant trend toward improved sprint performance [SMD = 0.37, 95% CI (−0.07, 0.80), P = 0.10]. In the moderate-rest subgroup (8 effect sizes), heterogeneity was also high (I² = 70%, P = 0.001); thus, a random-effects model was used, and the pooled effect indicated no significant improvement [SMD = 0.14, 95% CI (−0.34, 0.63), P = 0.56]. In the long-rest subgroup (15 effect sizes), heterogeneity was low (I² = 32%, P = 0.11); therefore, a random-effects model was used, and the pooled effect showed a significant improvement in sprint ability [SMD = 0.42, 95% CI (0.16, 0.68), P = 0.002]. Overall, these findings suggest that longer rest intervals may yield more consistent acute sprint benefits, whereas short and moderate rest intervals show highly variable responses.

### 3.8 Acute influence of plyometric training on sprint performance across different sprint distances (≤30 m *vs*. >30 m)

**Figure 8:**
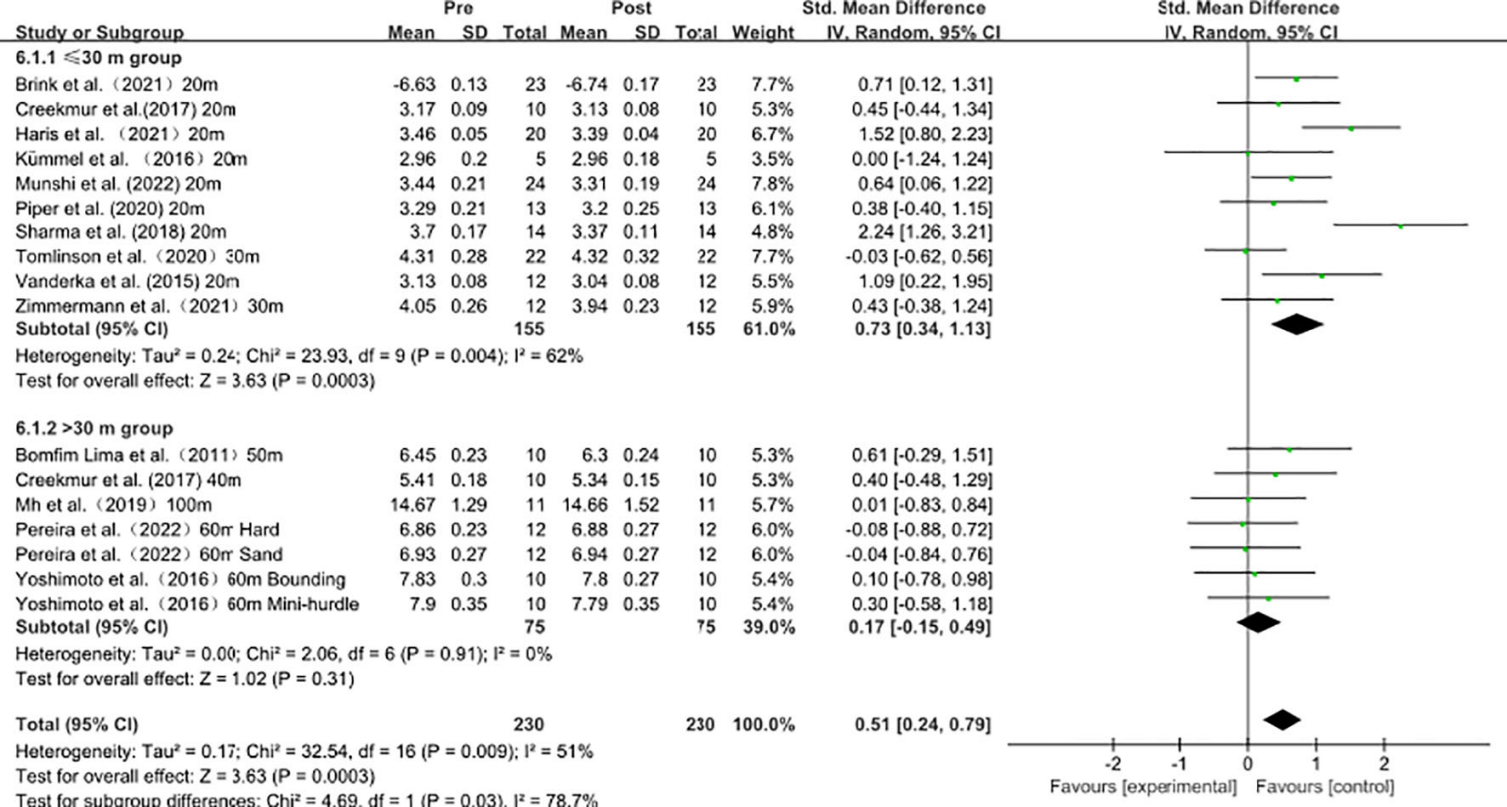
Acute influence of plyometric training on sprint performance across different sprint distances (≤30 m vs. >30 m).

As shown in **Figure 8**, subgroup analyses were performed to examine whether sprint distance moderates the effects of plyometric training on sprint performance (≤30 m *vs*. >30 m). In the ≤30 m group (10 effect sizes), heterogeneity analysis revealed moderate-to-high heterogeneity (I² = 62%, P = 0.004); therefore, a random-effects model was applied. The pooled effect indicated a significant improvement in short-distance sprint performance [SMD = 0.73, 95% CI (0.34, 1.13), P = 0.0003].

In contrast, in the >30 m group (7 effect sizes), no statistical heterogeneity was observed (I² = 0%, P = 0.91); a random-effects model was used, and the pooled effect was small and not statistically significant [SMD = 0.17, 95% CI (−0.15, 0.49), P = 0.31].

A significant subgroup difference was observed (χ² = 4.69, P = 0.03), indicating that sprint distance may be an important moderator of the acute effects of plyometric training on sprint performance.

## 4 Discussion

### 4.1 Acute influence of plyometric training on sprint performance

The meta-analysis suggests that plyometric conditioning activities (CAs) centered on jumping and reactive actions may be associated with improvements in sprint output within a short post–warm-up time frame. This phenomenon may be better framed within the broader construct of post-activation performance enhancement (PAPE), rather than being restricted to the classical concept of post-activation potentiation (PAP). One plausible explanation is that performance improvements induced by plyometric CAs are unlikely to be governed by a single mechanism: they may reflect PAP-related increases in neuromuscular excitability and myosin regulatory light-chain phosphorylation, while also integrating multiple pathways that commonly accompany warm-up, including elevations in muscle temperature, improved local perfusion, alterations in muscle viscoelastic properties, and heightened perceived arousal (Blazevich and Babault, 2019; Boullosa et al., 2020). From a warm-up physiology perspective, the rise in muscle temperature elicited by active warm-up may increase nerve conduction velocity and improve the efficiency of contraction kinetics, while potentially facilitating oxygen uptake kinetics and the onset of energy provision, thereby placing athletes in a more favorable preparatory state for high-power tasks (Bishop, 2003). In high-level competitive settings, evidence from inter-bout or pre-competition “re-warm-up” studies further suggests that incorporating brief activities with intensities closer to sport-specific demands may help maintain or restore sprint-related capacities (Abade et al., 2017).

Within a systematic warm-up structure, plyometric CAs may serve as a form of specific activation: they impose minimal time cost while rapidly recruiting high-threshold motor units and organizing rhythmic neural drive, which may contribute to a higher level of neuromuscular readiness for subsequent sprint acceleration (McGowan et al., 2015). Because plyometric CAs rely heavily on the stretch–shortening cycle (SSC), their acute effects may also operate through enhanced pre-activation, increased effective stiffness of the muscle–tendon complex, and improved elastic energy storage and return, which could facilitate force production during ground contact (Komi, 2000). Sprint performance—particularly during the acceleration phase—is strongly influenced by the capacity to generate force rapidly within a narrow time window; in explosive tasks, changes in the early rate of rise of the force–time curve may be more closely related to performance than maximal force (Maffiuletti et al., 2016). From a practical standpoint, it should be noted that excessive reliance on static stretching during warm-up may impair sprint performance; therefore, in contexts where sprint outcomes are primary, dynamic stretching and plyometric CAs may be prioritized to support the terminal activation phase of warm-up (Behm and Chaouachi, 2011).

### 4.2 Acute influence of single plyometric training on sprint performance

The meta-analysis indicates that a single-mode plyometric CA yields a more stable and reproducible facilitation of sprint performance. This advantage may reflect a more streamlined stimulus structure and more controllable dosing, which together make it easier to complete sprint testing or enter competition within a time window where potentiation outweighs fatigue. At the neuromuscular level, jumping actions may reinforce inter-joint coordination and pre-activation patterns, enhance neural drive and rapid reflex-related modulation, and improve the availability of short-duration power output via efficient eccentric–concentric coupling within the SSC (Markovic and Mikulic, 2010). At the muscle–tendon complex level, plyometric actions—particularly reactive jumps—are closely linked to stiffness regulation; mechanistic and training studies suggest that increases in stiffness and elastic energy storage/return can improve force-transmission efficiency, thereby providing a more favorable mechanical basis for force application during sprint ground contact (Kubo et al., 2007).

The greater stability of single-mode CAs may also be related to fatigue management. SSC-based models indicate that repeated jumping can elicit naturally occurring neuromuscular fatigue and fluctuations in motor control, whereas low-dose, short-duration plyometric stimuli are more likely to preserve (and potentially amplify) potentiation without disproportionately increasing the fatigue cost (Nicol et al., 2006). Because different loading schemes and recovery intervals can shift the subsequent trajectory of explosive output, the prescription precision of a single-mode CA (repetitions, intensity, and recovery) appears to directly determine its observable benefits (Chen et al., 2013). In addition, acute drop-jump protocols in competitive athletes have shown that appropriately designed single-bout jump stimuli can improve multiple indices of explosive performance, supporting their capacity to enhance performance under controlled fatigue conditions (Dello Iacono et al., 2016). In more ecologically valid settings, embedding a small number of jumps within the warm-up has also been associated with improved 20–40 m sprint performance (Creekmur et al., 2017). Finally, the relationship between drop-jump loading and subsequent performance may be moderated by individual force–velocity profiles, implying that CA load and exercise selection should be individualized in applied practice (Baena-Raya et al., 2020).

### 4.3 Acute influence of complex plyometric training on sprint performance

The meta-analysis suggests that complex plyometric CAs exhibit a stronger facilitative trend, albeit with greater variability in outcomes. From a training-theory perspective, complex plyometric CAs are conceptually analogous to the “complex training” paradigm, in which multi-component stimuli are combined and may amplify acute neuromuscular excitation and thereby provide a higher level of neural drive and kinetic-chain coordination for subsequent explosive tasks (Ebben, 2002). However, this paradigm also underscores the importance of sound pairing logic and clear prescription boundaries; in particular, careful control of exercise selection, total volume, and the recovery window is required to prevent fatigue from offsetting potentiation-related benefits (Docherty et al., 2004).

Relative to single-mode CAs, complex plyometric CAs are more likely to impose greater eccentric loading and mechanical impact, potentially providing a more robust neuromuscular stimulus. Such intensified stimuli may alter the ensuing potentiation response, but they also raise the threshold for fatigue management and expand the scope for inter-individual variability (Bridgeman et al., 2017). Accordingly, whether complex protocols translate into superior real-world performance may depend on whether the recovery interval is appropriately aligned with the time course over which potentiation becomes manifest. Evidence in professional rugby players indicates that different recovery arrangements can substantially modify the magnitude and direction of PAPE effects (Kilduff et al., 2007).

Individual training background is another major source of variability for complex plyometric CAs: athletes with higher training status or stronger strength foundations may better tolerate high-intensity stimuli and return more rapidly to a state conducive to high output. Early comparative work on PAP responses across training levels has likewise shown distinct response patterns between competitive athletes and recreationally trained individuals, consistent with the notion that complex protocols rely more heavily on high-threshold motor-unit recruitment and neuromuscular efficiency (Chiu et al., 2003). The importance of strength capacity is further supported by the premise that greater maximal and relative strength reduces the relative load imposed by a given stimulus, thereby increasing the likelihood of obtaining stable benefits from complex protocols (Suchomel et al., 2016; Tillin and Bishop, 2009).

### 4.4 Acute influence of complex plyometric training on sprint performance under different rest intervals

The meta-analysis indicates that, within the context of complex plyometric CAs, stratifying outcomes solely by recovery duration was insufficient to account for the observed variability. The inclusion of horizontally oriented jumps may represent one of several factors contributing to between-study differences; however, formal subgroup comparison is required to determine whether this factor represents a statistically confirmed moderator. From a mechanical standpoint, horizontal jumping is more aligned with the demands of the acceleration phase. Evidence suggests that an athlete’s capacity to effectively translate force into horizontal propulsion is a key mechanistic determinant of acceleration and short-sprint performance; accordingly, horizontal jumps are theoretically characterized by greater sprint specificity (Morin et al., 2011). Prior biomechanical syntheses further indicate that acceleration and maximal-velocity phases differ structurally in contact mechanics, force-vector orientation, and gait strategy, implying that CAs emphasizing distinct force directions may differentially influence phase-specific sprint outcomes (Mero et al., 1992).

However, greater sprint specificity does not necessarily translate into more stable net benefits, because complex protocols often elevate potentiation and fatigue concurrently. Findings in the sprint biomechanics literature indicate that higher running speed is driven primarily by greater ground reaction forces rather than increases in leg swing frequency; therefore, in high-intensity tasks with short ground-contact times, even small increments in fatigue may reduce available force and manifest as measurable decrements in speed (Rabita et al., 2015; Weyand et al., 2000). Consequently, when complex CAs incorporate horizontal jumps, the higher eccentric braking demands and greater execution-related variability may contribute to performance fluctuations that are difficult to explain by recovery duration alone. Classic physiological work has long established that potentiation and fatigue can coexist within the same neuromuscular system and that their decay kinetics are not synchronized; thus, if fatigue remains dominant at the time of testing, potentiation-related mechanisms may be effectively masked despite being present (Rassier and Macintosh, 2000). In addition, athletes with superior physical capacities and a higher proportion of fast-twitch fibers may be more capable of maintaining movement quality under high-impact, high-demand actions, thereby reducing outcome variability (Cronin and Hansen, 2005; Hamada et al., 2000).

### 4.5 Acute influence of plyometric training on sprint performance under different rest intervals

The meta-analysis suggests that longer recovery intervals (≥8 min) were associated with statistically significant pooled effects, whereas shorter and moderate recovery intervals did not reach statistical significance. However, statistical significance observed within individual subgroups does not necessarily indicate significant differences between subgroups. Formal tests for subgroup differences are required to determine whether recovery duration significantly moderates the effects of plyometric conditioning activities on sprint performance. Therefore, these findings should be interpreted with caution. Performance changes following a conditioning activity are highly dependent on the recovery schedule, because recovery dictates the rate at which metabolic fatigue, neural fatigue, and mechanically induced loading effects dissipate, thereby determining whether potentiation-related mechanisms can ultimately be expressed in performance outcomes (Gouvea et al., 2013; Hodgson et al., 2005). Evidence further suggests that fatigue is not a unitary phenomenon but rather the superposition of multi-level processes spanning the central nervous system to muscle fibers, and from neurotransmission to ionic homeostasis; consequently, the same nominal recovery duration may correspond to markedly different levels of functional restoration under different CA intensities and across athletes with different readiness states (Enoka and Duchateau, 2008).

At the cellular and molecular levels, fatigue-related mechanisms include constraints on Ca²^+^ release and reuptake, metabolite accumulation that perturbs cross-bridge function, and alterations in membrane excitability. These processes often require sufficient time to return toward baseline output capacity, which may contribute to variability in performance outcomes observed across different recovery durations, although the present meta-analysis does not provide sufficient statistical evidence that recovery duration independently moderates sprint performance (Allen et al., 2008; Xu et al., 2025). Practically, improving the usability of PAPE requires individualized pilot testing to determine each athlete’s optimal rest interval (Robbins, 2005; Simic et al., 2013). Accordingly, allocating a more ample recovery period after a plyometric CA may extend the warm-up benefit from a general temperature-mediated phase into a usable window dominated by sport-specific neuromuscular enhancement (Fradkin et al., 2010).

### 4.6 Acute influence of plyometric training on sprint performance across different sprint distances (≤30 m *vs*. >30 m)

The meta-analysis indicated that plyometric CAs were associated with statistically significant pooled effects in shorter sprint distances (i.e., acceleration phase), whereas no significant pooled effects were observed in longer sprint distances. However, differences in statistical significance between subgroups do not necessarily indicate statistically confirmed subgroup differences, and formal subgroup comparison is required to determine whether sprint distance represents a true moderating factor. Acceleration performance depends on the ability to rapidly generate sufficient propulsive force and power within very short ground-contact times. Evidence suggests that maximal neuromuscular power is determined not only by force- and velocity-related capacities, but also by the efficiency of recruiting high-threshold motor units (Cormie et al., 2011). In parallel, plyometric and other speed–strength actions specifically target rapid force production and short-contact output, which may help explain why significant pooled effects were observed in short-distance sprint performance (Cormie et al., 2011; Delecluse, 1997; De Oliveira et al., 2017). Reviews further indicate that PAPE responses in sprinting and jumping are jointly moderated by strength level, stimulus intensity, and the recovery interval; therefore, short sprints may be more likely to show measurable gains when recovery timing is optimized (Seitz and Haff, 2016).

Although this work focuses on acute effects, long-term training evidence similarly shows that plyometric training has been associated with improvements in sprint performance, particularly in acceleration-related measures, indirectly supporting a stronger coupling between plyometric-related capacities and acceleration performance (Saez de Villarreal et al., 2012). Classic intervention studies have also reported sprint improvements following plyometric training, consistent with substantial mechanical and neuromuscular commonalities between jumping and acceleration (Rimmer and Sleivert, 2000). By contrast, longer sprints depend more on gait stability at maximal speed and the ability to maintain velocity, which are additionally constrained by metabolic and muscle-tolerance factors. Because speed maintenance reflects more complex metabolic and muscular adaptations, a short PAPE window is likely to have limited influence on these processes; consequently, longer-distance total sprint times are less likely to show stable improvements (Ross and Leveritt, 2001).

### 4.7 Interpretation in the absence of control-group comparisons

In the absence of a control condition, it is difficult to disentangle observed PAPE-associated changes in performance from non-specific influences such as test familiarization (Glaister et al., 2010), trial-to-trial adjustments in pacing strategy, motivation and expectancy effects (Beedie and Foad, 2009), and circadian/diurnal fluctuations (Pullinger et al., 2020). In repeated sprint performance assessments, any apparent improvement may partly reflect general warm-up effects (e.g., increased muscle temperature, heightened arousal, and improved movement coordination) rather than a specific neuromuscular enhancement induced by the conditioning activity. Moreover, these changes may fall within normal diurnal variation and the typical error of the performance test, thereby limiting the strength of causal attribution in pre–post-only designs. In addition, PAPE responses show substantial inter-individual variability and are influenced by training status/strength level, muscle fiber phenotype, and genetic contributions to fiber-type distribution (Simoneau and Bouchard, 1995). Therefore, when rigorous within-participant control (e.g., randomized and counterbalanced crossover) or a parallel-group control is not implemented, attributing all performance improvements solely to PAPE should be regarded as a cautious and inferential interpretation. Future research should prioritize randomized crossover within-participant designs, counterbalancing, adequate familiarization, fixed testing time-of-day, and time-/activity-matched control or sham conditions to improve attribution of any net PAPE effect.

## 5 Conclusion

Overall, plyometric conditioning activities were associated with acute improvements in sprint performance, although responses varied across protocols and participants. Single plyometric conditioning activities were associated with small but relatively consistent improvements, supporting their practical use as an efficient warm-up strategy. Complex plyometric conditioning activities were associated with larger pooled effect sizes but also greater variability, highlighting the importance of appropriate exercise selection, recovery timing, and individualization. Rest interval and protocol characteristics appeared to influence responsiveness, with longer rest durations and specific exercise configurations showing more consistent pooled effects. Sprint-distance stratification showed statistically significant pooled effects in shorter sprint distances (≤30 m), whereas responses in longer distances (>30 m) were smaller and more variable. These findings align with the mechanical and neuromuscular characteristics of plyometric activities, which closely relate to acceleration performance.

### 5.1 Practical application

Coaches and athletes may consider incorporating plyometric conditioning activities into warm-up routines as a potential strategy to support neuromuscular readiness prior to sprint performance. Plyometric drills generally require minimal equipment and can often be implemented using body weight, which may facilitate their use across a wide range of sport settings. Based on the findings of this review, several practical considerations may be relevant for athletes with prior plyometric experience. For sprint tasks emphasizing acceleration (≤30 m), when warm-up time is limited or competition schedules are constrained, coaches and athletes may consider performing 2–4 sets of single plyometric conditioning activities, followed by a recovery period of approximately 8–16 min. When sufficient time and individual readiness permit, incorporating 2–4 sets of more complex plyometric conditioning activities with a similar recovery duration could also be considered, depending on athlete preference and tolerance. For sprint tasks exceeding 30 m, responses to plyometric conditioning activities appeared more variable. In these situations, coaches and athletes may consider maintaining a relatively low plyometric volume and combining plyometric exercises with sprint-specific drills, while allowing adequate recovery before maximal sprint efforts.

### 5.2 Research deficiencies and prospects

Although plyometric conditioning activities were associated with acute changes in sprint performance, several limitations should be acknowledged. First, the number of included studies and participants was relatively small (14 studies, 198 participants), which may limit the generalizability and precision of the pooled estimates. Second, substantial heterogeneity in protocol characteristics, including jump type, loading, volume, and recovery duration, may have contributed to variability in outcomes. Additionally, sprint testing procedures and contextual factors were not consistently reported, and participant characteristics, particularly sex distribution, were often imbalanced.

Future studies using adequately powered randomized or crossover designs, standardized sprint testing, and more diverse participant samples may help provide clearer guidance for practice.
